# Exaggerated Sexual Swellings and the Probability of Conception in Wild Sanje Mangabeys (*Cercocebus sanjei*)

**DOI:** 10.1007/s10764-017-9961-1

**Published:** 2017-04-25

**Authors:** David Fernández, Diane Doran-Sheehy, Carola Borries, Carolyn L. Ehardt

**Affiliations:** 1 Interdepartmental Doctoral Program in Anthropological Sciences, Stony Brook University, Stony Brook, NY 11794 USA; 2 Department of Applied Sciences, University of the West of England, Bristol, BS16 1QY UK; 3 Department of Anthropology, Stony Brook University, Stony Brook, NY 11794 USA; 4 Department of Anthropology, University of Texas at San Antonio, San Antonio, TX 78249 USA

**Keywords:** Adolescent, Estradiol, Paternity confusion, Postconceptive swelling, Sexual conflict

## Abstract

Females of several catarrhine primate species exhibit exaggerated sexual swellings that change in size and coloration during the menstrual cycle and, in some species, gestation. Although their function remains under debate, studies indicate that swellings may contain information males could use to discern ovulation and the probability that a cycle will be conceptive. Here we combine visual ratings of swellings with hormonal data for a group of Sanje mangabeys (18 adult, 3 adolescent females) to determine if their swellings provide reliable information on female fertility. In all cases where ovulation was detected (*N* = 7), it occurred during maximum tumescence, and in 83.3% during the first two days of the “shiny phase,” a period during maximum tumescence when the swelling was brightest. There were no significant differences in maximum tumescence and shiny phase duration among cycles of different probability of conception, although there was a trend toward conceptive cycles exhibiting shorter shiny phases than nonconceptive ones. Only 25% (*N* = 4) of postconceptive swellings developed the shiny phase, and adolescents displayed the longest maximum tumescence and shiny phases. The conspicuous nature of the shiny phase and the frequent overlap between its onset and ovulation suggest that its presence serves as a general signal of ovulation and that the cycle has a high probability of being conceptive. It also suggests that swellings in some Sanje mangabeys are more accurate signals of fertility than in other primates.

**Electronic supplementary material:**

The online version of this article (doi:10.1007/s10764-017-9961-1) contains supplementary material, which is available to authorized users.

## Introduction

Females of several catarrhine primates exhibit exaggerated sexual swellings that change in size and coloration during the menstrual cycle, as well as with female age and reproductive state (Dixson 2012). In these species, the skin around the vulvar and/or anal region swells and shrinks in response to fluctuations in ovarian hormone levels, peaking in size around the time of ovulation. In particular, estradiol induces tumescence, while progesterone triggers detumescence (Gillman 1940; Zuckerman 1937). Such sexual swellings are associated with multimale mating systems, in which females may have multiple mating partners, and with species that lack a distinct breeding season (Clutton-Brock and Harvey 1976; Nunn 1999; van Schaik *et al.* 2001).

Several hypotheses have been proposed to explain the function of sexual swellings (the best-male hypothesis: Clutton-Brock and Harvey 1976; the many-male hypothesis: Hrdy 1981; Hrdy and Whitten 1987; the obvious-ovulation hypothesis: Hamilton 1984; the male services hypothesis: van Noordwijk 1985; the reliable indicator hypothesis: Pagel 1994; the graded-signal hypothesis: Nunn 1999; the differentiating between cycles hypothesis: Emery and Whitten 2003; Zinner *et al.* 2002; the paternal care hypothesis: Alberts and Fitzpatrick 2012; see Nunn 1999 for a review). Although these hypotheses may differ in the benefits that sexual swellings provide to females, they are not mutually exclusive and often address different levels of variation in swelling size (Alberts and Fitzpatrick 2012). For example, most hypotheses address variation within a single cycle, typically whether maximum swelling size signals the timing of ovulation within the menstrual cycle (the best-male hypothesis, the many-male hypothesis, the obvious-ovulation hypothesis, the male services hypothesis, the graded-signal hypothesis: Nunn 1999). Other hypotheses consider whether variation in swelling size between cycles of the same female conveys information on cycle quality, such as the probability of conception (the differentiating between cycles hypothesis: Emery and Whitten 2003; Zinner *et al.* 2002). Finally, some hypotheses suggest that variation in swelling size provides information about the relative quality, e.g., physical condition, of females (the reliable indicator hypothesis: Pagel 1994).

Hypotheses focusing on variation in sexual swelling size within a menstrual cycle propose that swellings indicate when ovulation occurs and thus they predict that ovulation will coincide with maximum tumescence, when the swelling is largest. Accordingly, most studies that examined the relationship between ovulation and swelling size found that, although not always for every cycle, ovulation tended to occur during peak swelling, normally just prior to detumescence: olive (*Papio anubis*) and chacma (*P. cynocephalus*) baboons: Daspre *et al.* 2009; Higham *et al.* 2008a, b; Shaikh *et al.* 1982; Wildt *et al.* 1977; sooty mangabeys (*Cercocebus atys*: Aidara *et al.* 1981; Whitten and Russell 1996); bonobos (*Pan paniscus*: Reichert *et al.* 2002); chimpanzees (*P. troglodytes verus*: Deschner *et al.* 2003, 2004; Emery and Whitten 2003); long-tailed macaques (*Macaca fascicularis*: Engelhardt *et al.* 2005); Barbary macaques (*M. sylvanus*: Brauch *et al.* 2007; Möhle *et al.* 2005); crested macaques (*M. nigra*: Higham *et al.* 2012); reviewed in Street *et al.* (2016). In some species, however, such as bonobos (Douglas *et al.* 2016; Reichert *et al.* 2002) or long-tailed macaques (Engelhardt *et al.* 2005), this relationship is not as closely linked, and sexual swellings are not as reliable signals of ovulation. This interspecific variation in the reliability of sexual swellings as ovulatory signals may reflect changes in females’ mating strategies as a response to varying social systems and/or ecological variables. Such changes would ensure that females express their mating preference despite males’ own mating strategies. For instance, the long maximum tumescence durations and relatively low predictability of bonobos’ sexual swellings could hinder males’ mating strategies by reducing their ability to monopolize females (Douglas *et al.* 2016).

There is also growing evidence that variation in sexual swelling size between cycles relates to the female’s probability of conception. For example, some studies have shown that as females undergo menstrual cycling following a period of postpartum amenorrhea, ovarian hormone levels increase gradually so that later cycles are more likely to be conceptive than earlier ones (Emery and Whitten 2003; McCabe *et al.* 2013). Accordingly, as female baboon and chimpanzees cycle and their probability of conception increases, their sexual swelling size also increases (Deschner *et al.* 2004; Emery and Whitten 2003; Fitzpatrick *et al.* 2014; Higham *et al.* 2008b; Huchard *et al.* 2009), with those in conceptive cycles being significantly larger than those in nonconceptive ones (Alberts *et al.* 2006; Daspre *et al.* 2009; Gesquiere *et al.* 2007; Higham *et al.* 2012). Since males prefer mating with females that undergo conceptive cycles (Alberts *et al.* 2006; Bulger 1993; Daspre *et al.* 2009; Engelhardt *et al.* 2004; Gesquiere *et al.* 2007; Weingrill *et al.* 2003), this variation in male preference suggests that sexual swellings may convey information about the quality of the cycle and thus the probability of conception.

In several primate species, females also develop sexual swellings and mate during gestation, e.g., pig-tailed macaques (*M. nemestrina*: Hadidian and Bernstein 1979); chimpanzees (Wallis 1982); long-tailed macaques (van Noordwijk 1985); Tana river mangabeys (*C. galeritus*: Kinnaird 1990); sooty mangabeys (Gordon *et al.* 1991); Barbary macaques (Möhle *et al.* 2005). Given that conception is not possible at this time, it is possible that postconceptive mating functions to further confuse paternity among males, thereby reducing the risk of infanticide and increasing paternal investment (Hrdy 1979; Hrdy and Whitten 1987; cf. Doran-Sheehy *et al.* 2009 and reference therein for additional explanations). This would be an effective strategy, however, only if males are unable to distinguish between the sexual swellings of pregnant and nonpregnant females, which does appear to be the case in some studies (Barbary macaques: Small 1990; long-tailed macaques: Engelhardt *et al.* 2007), but not in others (Barbary macaques: Küster and Paul 1984; sooty mangabeys: Gordon *et al.* 1991; Gust 1994). In sooty mangabeys, for example, although the only difference that exists between postconceptive swellings and swellings developed during menstrual cycling is that the former swellings take longer to deflate (Gordon *et al.* 1991), males show preference for maximally tumescent cycling females over pregnant females with postconceptive swellings (Gust 1994).

Finally, variation in sexual swelling size between cycles may also advertise differences in fertility between very young and more mature females, as adolescent females usually undergo a period of infertility often characterized by irregular, anovulatory cycles (Hartman 1931; reviewed in Dixson 2012). Adolescents of several primate species, however, display exaggerated versions of adults’ fertility cues, including swellings that are larger and more brightly colored than those of adults (Anderson and Bielert 1994). Therefore, in contrast to what happens in adults, sexual swelling size in adolescent females does not seem to correlate with the female’s probability of conception. Instead, size may function to overcome males’ lower preference toward adolescent females (Anderson and Bielert 1994).

The majority of research on sexual swellings has been on apes, macaques and baboons, and the only studies on mangabeys to date have been on captive populations (Aidara *et al.* 1981; Gust 1994; Walker *et al.* 2004; Whitten and Russell 1996). Here, we address this taxonomic imbalance by investigating the function of sexual swellings in wild Sanje mangabeys (*C. sanjei*), an African cercopithecine that develops exaggerated sexual swellings during both the menstrual cycle and gestation. In particular, we use visual ratings of swellings and hormonal data to examine 1) the relationship between intracycle variation in swelling size and the time of ovulation; and 2) the relationship between intercycle variation and the probability of conception. Because currently there are descriptions of sexual swellings only for two other species of mangabeys (sooty mangabey: Aidara *et al.* 1981; Whitten and Russell 1996; golden-bellied mangabey: Walker *et al.* 2004), we first present a quantitative description of Sanje mangabey’s cycle length and the duration of maximum tumescence. We also describe a characteristic “shiny phase” of the swelling that occurs during most cycles for this species. To examine the extent to which sexual swellings accurately signal ovulation, we identify the timing of ovulation using hormonal data, i.e., fecal estradiol metabolite concentrations (fE) and compare its timing to that of maximum tumescence and the shiny phase. We use these same characteristics, i.e., duration of maximum tumescence and duration of the shiny phase, to test the hypothesis that differences between sexual swellings predict the probability of conception (maximum, high, low or zero) for a given cycle.

## Methods

### Study Site and Subjects

We conducted this research on a habituated group of Sanje mangabeys inhabiting the Mwanihana Forest (7**°**40′–7**°**57′S, 36**°**46′–36**°**56′E) of the Udzungwa Mountains National Park, Tanzania. The habitat is a mosaic of montane and submontane tropical forest, interspersed by areas of deciduous primary and secondary vegetation (Ehardt *et al.* 2005). Annual rainfall in the region averages 1750 mm (Lovett 1996), of which 90% falls during the rainy season from November to May (McCabe and Emery Thompson 2013).

Sanje mangabeys exhibit a polgynandrous mating system, in which males are dominant to females and dominant males typically mate-guard sexually receptive females (Fernández 2016). Matings and births occur throughout the year; however, most (64%) conceptions occur between January and March (McCabe and Emery Thompson 2013). The study group, the Mizimu group, was first habituated in 2004 and has been monitored regularly ever since (Ehardt *et al.* 2005). During the study, the group consisted of 63–65 individuals, including 7–10 adult males, 18–20 adult females, 3 adolescent females, and juveniles and infants. We identified all adult and cycling adolescent females using scars and facial coloration. We distinguished adolescent females from adults by the smaller body size, button-like nipples (Altmann *et al.* 1977), and lighter colored facial skin of the latter. Adolescents underwent a period of infertility (Dixson 2012; Hartman 1931), cycling for up to 16 mo without conceiving (Fernández *et al.* 2014). Data presented here are from 18 adult and 3 adolescent females.

### Data Collection

D. Fernández and a team of assistants collected the data for this study during two periods. During period 1 (October 13, 2008–May 1, 2009), we collected observational data on sexual swelling size and color and female reproductive state. In period 2 (June 11, 2009–July 10, 2010), we added fecal collection for hormonal analysis to the observational protocol. During period 1, we followed the group for a mean of 8.8 ± SD 4.3 days/mo (range: 4–18, total = 70 days), while during period 2 we followed it a mean of 23.1 ± SD 6.0 days/mo (range: 10–30 days), for a total of 338 days or 3346.4 h (mean = 10.36 ± SD 1.2 h/observation day). We typically stayed with the group (*N* = 408 follows) from sleeping site to sleeping site, with the exception of 2 days during period 2, in which we lost contact with the group after 1.3 and 1.6 h, respectively.

#### Sexual Swelling Scores

We began collecting data on sexual swelling size during period 1, once we reliably identified all adult and adolescent females (December 1, 2008 onward) and continued for the duration of the study. To ensure accuracy of swelling score data, observers discussed their individual assessment on arrival back at camp each day. We began collecting data on presence of the shiny phase in September 2009, when it had become clear that this characteristic was a very conspicuous trait. Each day we followed the group we collected sexual swelling size using a 9-point visual scale, modified after Whitten and Russell (1996) and Walker *et al.* (2004). Swellings were then categorized into one of four states including a) the absence of any sexual swelling (score 0); 2) a sexual swelling increasing in tumescence, i.e., inflation (scores 1**–**4); c) maximum tumescence (score 5); or d) a sexual swelling decreasing in tumescence, i.e., detumescence or deflation (scores 6**–**8) (Table I; Electronic Supplementary Material [ESM] Fig. S1). We designed the scale to capture progressive changes in the tumescence of the swelling rather than its absolute size; therefore, two females may have the same swelling score, e.g., score 5, but swellings that differ in their absolute size. In addition to recording swelling size, during each day of maximum tumescence we scored the presence or absence of the shiny phase, which gave the swelling a shinier, brighter tone. We did not observe any systematic changes in the coloration of the sexual skin.

**Table I:**
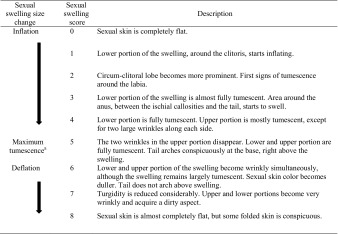
Description of the nine stages used to code changes in sexual swelling size in Sanje mangabeys during the menstrual cycle and gestation

The scale was modified after Whitten and Russell (1996) and Walker *et al.* (2004)

^a^The shiny phase occurs during size 5. See text for further details

#### Female Reproductive State

We distinguished three reproductive states (cycling, pregnant, and postpartum amenorrhea) based on changes in sexual swelling size and on daily records of infant births and deaths. As in other *Cercocebus* species (Hadidian and Bernstein 1979; Walker *et al.* 2004; Whitten and Russell 1996), cycling females underwent successive inflations and deflations, reaching maximum tumescence ca. every 30 days (Fernández *et al.* 2014), and pregnant females developed a postconceptive swelling that peaked in size ca. 50 days after conception (Gordon *et al.* 1991; Walker *et al.* 2004). We used these characteristics to identify conceptions and pregnant females (Fernández *et al.* 2014). All conceptions were eventually confirmed hormonally when such data were available (*N* = 3 conceptions) and/or with the subsequent parturition ca. 172 days after conception, the mean gestation length for this species (Fernández *et al.* 2014). We classified females as being in postpartum amenorrhea from the day of parturition until they developed the first sexual swelling postpartum. We excluded data collected from late March to early July 2010; a period when eight of nine cycling adult females and one adolescent female stopped cycling regularly and exhibited significantly different concentrations of fE metabolites (Fernández *unpubl. Data*), possibly due to the seasonal consumption of phytosteroids (Emery Thompson *et al.* 2008; Higham *et al.* 2007; Lu *et al.* 2011).

#### Fecal Sample Collection and Hormone Assays

To approximate the day of ovulation we collected fecal samples during period 2 in order to capture the estrogen surge that precedes ovulation (Fernández *et al.* 2014; Saltzman *et al.* 2010). We also analyzed fecal samples for progesterone levels to identify the postovulatory progesterone rise that occurs after ovulation (Saltzman *et al.* 2010). As described elsewhere, however, the progestogen assay we used was not able to reliably detect ovulation in this species from fecal samples (Fernández *et al.* 2014). In total we analyzed 936 samples, including 735 from adult females (mean = 31.0 ± SD 19.8 samples/female, range: 3–71 samples) and 201 from adolescent females (mean = 67.0 ± SD 12.2 samples/female, range: 53–75 samples). On average, we sampled adult cycling females a mean of 1.9 ± SD 0.9 days (range: 1–5 days) when they were approaching, i.e., reached swelling size score 4, or in maximum tumescence, and every 4.5 ± SD 2.1 days (range: 1–12 days) outside maximum tumescence. Additionally, we sampled adolescent females every 2.3 ± SD 1.0 days (range: 1–8 days) when approaching or in maximum tumescence, and 5.2 ± SD 3.1 days (range: 1–20 days) outside this period.

We followed Brockman and Whitten (1996) for the collection and preservation of feces. We collected recently deposited samples that had not been contaminated with urine using plastic bags containing silica gel. We maintained feces cool for 2–8 h, until dried in a Coleman® oven. We then stored the dried feces in labeled Ziploc® bags with silica gel until shipped to the Smithsonian’s National Zoological Park, Front Royal, VA, for analysis. Fecal extraction protocols followed Fernández *et al.* (2014). Briefly, we lyophilized, sifted, and stored samples in 5-ml polypropylene tubes until analyzed. Mean fecal extraction efficiency was 76.4 ± SD 15.2% based on recovery of radiolabeled steroid added to samples before extraction. We analyzed the extracts for fE metabolites using enzyme immunoassay procedures. The antiserum (R4972; provided by C. Munro, UC Davis) had cross-reactivities of 100% with estradiol, 3.3% with estrone, 0.8% with progesterone, 1.0% with testosterone and androstenedione, and <1% with cortisol and dihydrotestosterone (J. L. Brown *pers. comm.*). Mean recovery of exogenous steroid before extraction was 93.3 ± SD 8.1% (*N* = 2/hormone). Assay sensitivity was 40 pg/ml. Interassay coefficient of variations (*N* = 32) for low and high controls were 8.3% and 8.8%, respectively, while intraassay CVs were 7.4% for high and 8.0% for low controls.

### Data Analysis

#### Detection of Ovulation

We restricted the analysis of ovulation to those cycles where we collected hormonal samples a minimum of 50% of the days during maximum tumescence, the period when ovulation was most likely to occur (Aidara *et al.* 1981; Whitten and Russell 1996). We used the methods described in Fernández *et al.* (2014) to detect ovulation. First, we calculated a baseline value of fE using an iterative process during which we repeatedly excluded all values exceeding 1.5*SD above the mean (Brown *et al.* 1996). We averaged the remaining values to calculate the fE baseline. Next, we calculated the fE surge threshold, defined as 1.5 times above the baseline. Finally, we identified all fE peaks, i.e., those exceeding the fE surge threshold. In the related *C. atys*, levels of fE correlate with levels of serum estradiol (Whitten and Russell 1996), which in turn correlate with levels of follicle-stimulating hormone (Aidara *et al.* 1981). Thus, we used the fE peak as a proxy for the ovulatory surge of serum estradiol that occurs prior to ovulation (Fernández *et al.* 2014). When there were no hormonal data the day immediately before and/or after the fE peaks, we could not discard the possibility that the fE levels also exceeded the fE baseline on those days. Thus, to be conservative we included either of those days before and/or after the fE peak as part of the peak. Therefore, in these cases, the estrogen surge was an “ovulatory window,” which included the day(s) that fE rose above the threshold, plus the day(s) without samples on either side of it (details in Fernández *et al.* 2014). Given that in *C. atys*, serum estradiol was metabolized and appeared in feces within 0–2 days (Whitten and Russell 1996), we used a 24-h time lag to account for metabolism. Ovulation, however, occurs 24 h after serum estradiol peaks (Jeffcoate 1983); thus, we used the day of the estrogen surge as the day fE exceeded the baseline in feces (Higham *et al.* 2008b).

We identified eight adult female cycles that were well sampled during and around maximum tumescence, including four nonconceptive, three conceptive, and one anovulatory cycle. One of these cycles occurred before September 2009; thus, we had not coded the presence of the shiny phase. For adolescent females, we identified three ovulatory, nonconceptive cycles.

#### Changes in Sexual Swellings during Cycling

To describe the changes in sexual swelling we calculated the following parameters of the cycles of adult females: 1) cycle length (from the first day of detumescence to the last day before the onset of the next detumescence); 2) inflation (from the first day the swelling starts to increase in size after reaching its minimum score, until the last day before reaching maximum tumescence); 3) maximum tumescence (the number of days the sexual swelling was at maximum size; i.e., score 5); 4) the shiny phase (the number of days during maximum tumescence with a shiny appearance); 5) deflation (from the first day of detumescence to the last day before the swelling reaches its minimum score); and 6) minimum swelling (the number of days during which the swelling remained at its minimum score). We differentiated between conceptive and nonconceptive cycles in the aforementioned calculations, as studies of some species have shown that swellings during conceptive cycles are larger than swellings during nonconceptive cycles (Alberts *et al.* 2006; Daspre *et al.* 2009; Gesquiere *et al.* 2007; Higham *et al.* 2012). We also determined whether the duration of maximum tumescence in adult females correlated with the duration of the shiny phase. To do so, we used only cases for which the exact duration of both was known (*N* = 12). We excluded from analyses all cases when the changes in sexual swelling size deviated from the regular pattern of inflation – maximum tumescence – shiny phase – deflation (*N* = 8, typically females resuming cycling after postpartum amenorrhea; ESM Table SI).

#### Changes in Sexual Swellings in Relation to Ovulation

To assess the relationship among maximum tumescence, the shiny phase, and ovulation, we plotted each ovulatory cycle aligned to the first day of detumescence.

#### Changes in Sexual Swellings in Relation to the Probability of Conception

To determine if sexual swellings convey information on the probability of conception for a given cycle we compared the duration of maximum tumescence and the shiny phase across cycles of different fertility. First, we tested for differences in these two characteristics between swellings of maximum, i.e., swellings developed during conceptive cycles, and high probability of conception, i.e., swellings developed during nonconceptive cycles, including the first cycle after infant death (Altmann *et al.* 1978; Higham *et al.* 2009). When we did not find significant differences in the duration of maximum tumescence and/or the shiny phase between swellings of maximum and high probability of conception, we pooled these data and tested them against swellings developed during low, i.e., first cycle after postpartum amenorrhea following a surviving infant and cycles of adolescent females, and zero, i.e., postconceptive swellings, probability of conception. If swellings in Sanje mangabeys accurately convey information on the probability of conception we expect that the duration of maximum tumescence and/or the shiny phase will vary according to the fertility of the cycle.

### Data Treatment

To examine the characteristics of the sexual swelling tumescence and appearance we limited our calculations to instances in which we knew the exact start and end day of each characteristic. We also examined all cases (*N* = 34) when we could determine the start and end date within 1 or 2 days. We report the latter values only when they fall outside the range of variation observed in the data set with the stricter criterion. Before analysis, we screened our data for equal variance, normal distribution, and outliers. As our sample size was small, we used each measure, e.g., maximum tumescence duration, rather than each female, as the unit of analysis (Lu *et al.* 2010). We also undertook statistical tests using the mean for each female, as a female may contribute more than once to each dataset, rendering the data not independent. In most cases, we confirmed the results; thus we report results based on individual values. We also add results based on mean values only if they differed. We identified outliers, defined as values whose distance from the nearest quartile was greater than 1.5 times the interquartile range, using the Outlier function in SPSS 19.0 for Mac and were. We conducted statistical analyses in R 3.2.3 (R Development Core Team 2015) for Mac. We assessed differences in the duration of maximum tumescence and the shiny phase between sexual swelling types and between adult and adolescent females via Mann–Whitney U tests using the Coin package (Hothorn *et al.* 2008). All tests were two-tailed and evaluated with an α level of 0.05. Tests with a *P* value >0.05 but <0.1 are reported as statistical trends.

### Ethical Note

Methods used in this study did not affect the welfare or the behavior of the study animals, and complied with protocols approved by the IACUC at Stony Brook University (2006–2010/1559) and by Tanzanian government authorities.

## Results

### Changes in Sexual Swellings during Cycling

Average cycle length was significantly shorter in adult vs. adolescent females (nonconceptive cycles: Mann–Whiney *U*: *z =* 2.732, *P* = 0.002; Conceptive cycles: Mann–Whiney *U*: *z* = 2.323, *P* = 0.029; Table II). The significant difference with nonconceptive cycles was a statistical trend when we used a mean for each female (one-sample Wilcoxon test: *V* = 0, *P* = 0.063). Nonconceptive cycles of adult females included a mean period of inflation of 11.0 ± SD 5.2 days, a maximum tumescence of 6.3 ± SD 1.1 days, and a deflation of 5.6 ± SD 1.6 days (Table II). In conceptive cycles, mean inflation was 8.0 ± SD 3.5 days, maximum tumescence 1.7 ± SD 1.2 days, and deflation 20.3 ± SD 9.4 days. Typically, the sexual swelling did not deflate completely between the end of deflation and the start of subsequent inflation. Rather a minimum swelling (score 8–1) was maintained for a mean of 8.8 ± SD 0.8 days in nonconceptive cycles, and for 11.0 days in conceptive ones (Table II).

**Table II:** Changes in the sexual swellings of Sanje mangabeys during their menstrual cycle

	Nonconceptive cycles								Conceptive cycles							
	Mean	SD	Median	SE	Min	Max	*N*	*N*_f_	Mean	SD	Median	SE	Min	Max	*N*	*N*_f_
Cycle length (adult)	29.4	1.9	29.0	0.6	27	32	8	5	28.0	2.8	29.0	1.2	24	30	4	4
Cycle length (adolescent)	44.5^a^	8.5	45.0	3.7	33	45	4	1	—	—	—	—	—	—	—	—
Inflation	11.0	5.2	9.0	2.1	6	19	5	3	8.0	3.5	10.0	1.63	4	10	3	3
Maximum tumescence	6.3	1.1	6.5	0.3	4	8	12	10	1.7	1.2	1.0	0.5	1	3	3	3
Deflation	4.3	1.3	4.0	0.5	3	6	4	3	20.3	9.4	17.0	4.5	13	31	3	3
Minimum swelling	8.8	0.8	9.0	0.3	8	10	5	3	11.0^b^	—	—	—	11	11	1	1

Data were collected in the Udzgunwa Mountains National Park from October 2008 through July 2010. All characteristics refer to adult females unless otherwise indicated. Duration given in days

*N* number of individual cases used for each calculation, *N*_f_ number of females that contribute to each parameter

^a^One case known to within 1 day lasted 22–23 days

^b^One case known to within 2 days maintained minimum swelling for 36–38 days

The mean duration of the shiny phase during nonconceptive cycles was 4.0 ± SD 1.7 days (Table III). It began 3.0 ± SD 1.7 days (range: 1–6 days, *N* = 9 swellings) after the swelling reached maximum tumescence, and ended a mean of 1.0 ± SD 0.7 days (range: 0–2 days; *N* = 9 swellings) before the onset of detumescence. The correlation between the duration of maximum tumescence and the shiny phased showed a positive statistical trend (Pearson correlation: *r* = 0.334, df = 9, *P* = 0.063, *N* = 12).

**Table III:** Characteristics of maximum tumescence (MAX) and shiny phase duration of sexual swellings of Sanje mangabeys during swelling of different conception probability

Swelling type	Conception probability	MAX (days)							Shiny phase (days)						
		Mean	SD	Median	SE	Min	Max	*N*	Mean	SD	Median	SE	Min	Max	*N*
Conceptive	Maximum	7.3	4.0	8.0	1.9	3	11	3	1.7	1.2	1.0	0.5	1	3	3
Cycling, nonconceptive^a^	High	6.3	1.1	6.5	0.3	4	8	12	4.0	1.7	4.0	0.5	1	6	11
First after PPA	Low	4.8	2.4	6.0	1.0	1	7^b^	5	3.3	1.5	3.0	0.7	2	5^c^	3
Adolescent female	Low	8.9	2.9	10.0	1.0	5	12	7	7.0	2.3	7.0	0.8	3	11	8
Postconceptive	Zero	7.0	1.4	7.0	0.7	6	8	2	0^d^	—	0	—	0	0	2

Data were collected in the Udzungwa Mountains National Park from October 2008 through July 2010. Only cases for which we had the exact start and end day of each characteristic were included. PPA = postpartum amenorrhea

^a^Combines ovulatory cycles and cycles without hormonal data

^b^One case known to within 2 days maintained MAX for 8–10 days

^c^One case known to within 2 days displayed the shiny phase for 8–10 days

^d^One case known to within 1 day displayed the shiny phase for 6–7 days

### Changes in Sexual Swelling in Relation to Ovulation

**Fig. 1:**
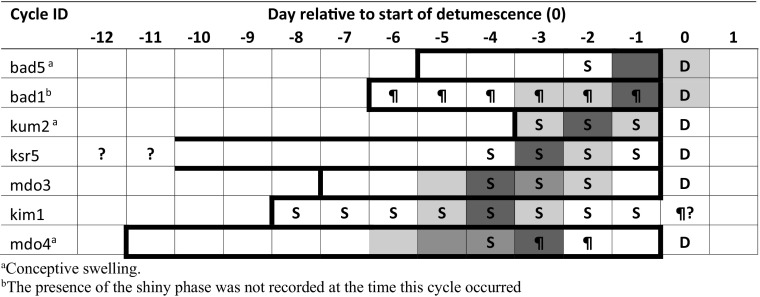
Changes in sexual swelling in relation to ovulation in a wild group of Sanje mangabeys inhabiting the Udzungwa Mountains National Park, Tanzania. Data were collected from October 2008 through July 2010. Maximum tumescence periods (bold outline) are aligned with respect to the day of detumescence (day 0). Dark gray boxes correspond to the highest fecal estradiol metabolite (fE) levels, indicative of the fE surge used as a proxy for ovulation; medium gray boxes correspond to lower fE levels but still above the fE threshold, and light gray boxes to days without fecal samples and thus without fE data. Cycles are ordered in terms of the proximity of the ovulatory window to the start of detumescence. S = the shiny phase; D = first day of detumescence; ? = unknown day of start of maximum tumescence or end of maximum tumescence; ¶ = no data on the presence or absence of the shiny phase.

In all cases where we had fecal hormones and swelling data, ovulation occurred during maximum tumescence (*N* = 7 cases; Fig. 1). In two cases, we could not rule out the possibility that ovulation occurred on the first day of detumescence, as there were no hormonal values on the first day of detumescence for those swellings (cycle ID: bad5 and bad1; Fig. 1). Ovulation never occurred before maximum tumescence. The first day of the shiny phase fell on the day of, or the day preceding, ovulation in five of six cases, including three conceptions (cycle ID: kum2, ksr5, mdo3, kim1, mdo4; Fig. 1). On average, the ovulatory window began a mean of 3.7 ± SD 1.7 days (range: 0–6) before the start of detumescence (Fig. 1).

### Changes in Sexual Swelling in Relation to the Probability of Conception

#### Maximum Probability of Conception

Sexual swellings during conceptive cycles remained at maximum tumescence for a mean of 7.3 ± SD 4.0 days, during which time they displayed the shiny phase for a mean of 1.7 ± SD 1.2 days (Table III).

#### High Probability of Conception

The average maximum tumescence duration during conceptive cycles did not differ significantly from nonconceptive cycles (*z* = 0.813, *P* = 0.488; Table III). When comparing the duration of the shiny phase during conceptive and nonconceptive cycles, however, we found a statistical trend (*z* = −1.909, *P* = 0.070; Table III). Therefore, to test for differences in maximum tumescence duration against swellings of lower probability of conception, we combined cases during maximum and high probability to allow for a more robust statistical comparison. Combined, maximum tumescence lasted a mean of 6.5 ± SD 1.9 days (median = 7.0 ± SE 0.5). We tested for differences in the duration of the shiny against swellings during maximum and high probability of conception separately, however, given that in conceptive cycles the shiny phase was less than half as long as in nonconceptive cycles (1.7 days vs. 4.0 days, respectively).

#### Low Probability of Conception

The duration of maximum tumescence of adult females during cycles with low probability of conception did not differ significantly from swellings during conceptive and nonconceptive cycles combined (*z* = 1.384, *P* = 0.170; Table III). Likewise, the duration of the shiny phase was not significantly different compared to the shiny phase displayed during swellings of conceptive or nonconceptive cycles (*z =* −1.348, *P* = 0.300 and *z =* 0.716, *P* = 0.539, respectively; Table III).

Similarly, duration of maximum tumescence of adolescent females’ swellings did not differ compared to maximum tumescence of adult females during cycles of maximum and high probability of conception (*z =* 1.684, *P* = 0.096; Table III). When using means for females this difference became significant (*z* = 2.575, *P* = 0.007), with adult females having longer maximum tumescence. In contrast, the duration of the shiny phase of adolescents was significantly longer than both adult conceptive (*z =* 2.403, *P* = 0.012; nonsignificant when using a mean for each female: *z* = 1.993, *P* = 0.100) and nonconceptive cycles (*z =* 2.702, *P* = 0.005), respectively (Table III).

#### Zero Probability of Conception

During gestation, all females developed a single postconceptive sexual swelling, which reached maximum tumescence a mean of 49.0 ± SD 1.4 days (range: 48–50 days, *N* = 3; extended dataset: 45–47 days) after the first day of detumescence of the conceptive cycle. One additional adult female displayed a swelling 55 days after the start of detumescence that reached only swelling score 4.

The duration of maximum tumescence in postconceptive swellings was not significantly different compared to maximum tumescence during conceptive and nonconceptive cycles combined (*z =* −0.533, *P* = 0.735; Table III). Postconceptive swellings were also less likely to display the shiny phase. Only one of the four postconceptive swellings that reached maximum tumescence displayed a shiny phase, which lasted 6–7 days. However, during all postconceptive swellings, we had one day without data; thus, we could not definitively exclude the possibility that the shiny phase was displayed that day.

## Discussion

Our analyses demonstrate that in wild Sanje mangabeys sexual swellings may convey information on female fertility. In this species, ovulation typically occurred during maximum tumescence, and particularly at the onset of the shiny phase, a period during maximum tumescence when the swelling became brightest. We did not find significant differences in the duration of maximum tumescence or in the shiny phase among cycles of different probability of conception. Sexual swellings during gestation, however, were less likely to develop the shiny phase, and compared to adult females adolescents displayed the shiny phase for longer. Thus, it seems that in adult females the presence of the shiny phase serves as a general indicator of the timing of ovulation and of the probability that a cycle will become conceptive.

The mean cycle length for adult Sanje mangabeys based on the swelling pattern was very similar to the mean cycle length calculated between successive menses (30.0 ± SD 3.0 days: Fernández *et al.* 2014), and slightly shorter but within the range reported for other *Cercocebus* (mean = 34.0 days: Stabenfeldt and Hendrickx 1973; median = 34.5 days: Hadidian and Bernstein 1979; mean = 30.8 days: Gordon *et al.* 1991; mean = 27.5–30.1 days: Whitten and Russell 1996; mean = 31.0 days: Walker *et al.* 2004) and the closely related *Mandrillus* (median = 33.5: Hadidian and Bernstein 1979; mean = 39.6 days: Bettinger *et al.* 1995; mean = 45 days: Setchell and Wickings 2004). Maximum tumescence duration for adults was also within the range of other *Cercocebus* (5–8 days: Whitten and Russell 1996; 2–12 days: Walker *et al.* 2004), although Aidara *et al.* (1981) reported shorter durations for a captive population of sooty mangabeys (2–3 days). In Sanje mangabeys, sexual swellings became brighter, i.e., the shiny phase, during maximum tumescence, a characteristic that has not been previously described for other *Cercocebus*, and thus there is no comparative reference value for this trait. More detailed studies on the sexual swellings of other *Cercocebus* mangabeys are necessary to elucidate the distribution of this trait or determine if it is unique to the Sanje mangabey.

Results of analyses of the timing of ovulation showed that ovulation occurred during maximum tumescence, typically during the second half, 3.7 days before the onset of detumescence. Furthermore, in five out of six cases the highest concentration of fE metabolites occurred during the first or second day of the shiny phase. There was some uncertainty, however, about the date of ovulation for four of the seven cycles used to examine the relationship between maximum tumescence and ovulation. In particular, there were several days around the presumed time of ovulation with no hormonal data (Fig. 1) that precluded us from ruling out whether ovulation occurred the first day of detumescence (cycle ID: bad5, bad 1) or 1 or 2 days before the onset of the shiny phase (cycle ID: mdo3, mdo4). Even if this were the case, however, results from the remaining cycles would still indicate that in the Sanje mangabey sexual swellings can convey information about ovulation, signaling when it is more likely to occur, i.e., during maximum tumescence, and particularly at the onset of the shiny phase.

We also compared the duration of maximum tumescence and the shiny phase to assess whether swellings can be used to predict the probability of conception. Overall, we found no robust evidence that either duration of maximum tumescence or the shiny phase was a reliable indicator of the probability of conception in a given cycle. There are two possible reasons why we may not have found significant differences. For one, we used a very conservative approach when deciding which data to include in the calculations, which rendered sample sizes fairly small, particularly for conceptive swellings. Such a small sample size may not have captured the whole range of variation that exists in the duration of maximum tumescence and/or the shiny phase—as values from the extended dataset seem to suggest (ESM Table SI)—thus, differences that may exist between different swelling types in these two characteristics may be masked in our analyses. More likely, however, is that the duration of maximum tumescence and the shiny phase per se are not indicators of cycle quality. Instead, it is plausible that in adult females the presence of the shiny phase itself, and not its duration, functions as a general signal for ovulation and that a cycle could be conceptive. Accordingly, the duration of maximum tumescence and the shiny phase may rather be a byproduct of the hormonal profiles of each cycle, and the shorter shiny phases displayed during conceptive cycles may indicate hormonal changes triggered by the fertilization of the ovum (Saltzman *et al.* 2010).

Taken together, our results suggest that the sexual swellings of Sanje mangabeys can potentially provide information to males on the timing of ovulation and the probability that a cycle will be conceptive. More specifically the presence of the shiny phase may serve as a general signal that indicates that females have reached the most fertile time of their cycle and that the cycle has a high probability of being conceptive. As such, males could use the shiny phase to identify when females are most likely to conceive, and invest their guarding and mating efforts in those females. If that is the case, we can make predictions as to which females males would prefer when more than one female is receptive at a time. First, males should prioritize females that exhibit maximum tumescence. In addition, among females at maximum tumescence, males should prefer to mate with those that have been at maximum tumescence for longer, as they are more likely to be in the second half of maximum tumescence, when ovulation tends to occur. Finally, males should select females that display the shiny phase, and particularly those that are as close to the onset of the shiny phase as possible, when ovulation typically happens. In humans, sperm is viable for up to 3 days (Wilcox *et al.* 1995), and since the ova is rarely viable for more than 24 h after its release (France 1981), any copulation that occurs any time from 3 days prior to ovulation, to the day after ovulation, can potentially lead to conception. Thus, to maximize the chances of fertilizing the ovum, male Sanje mangabeys should prioritize females from the time immediately before, through the time immediately after the start of the shiny phase.

Results from this study are in accordance with several hypotheses proposed to explain the function of exaggerated sexual swellings in primates, including the best-male hypothesis, the obvious-ovulation hypothesis, the many-male hypothesis, the male-service hypothesis, and the paternal care hypothesis (Alberts and Fitzpatrick 2012; Clutton-Brock and Harvey 1976; Hamilton 1984; Hrdy 1981; Hrdy and Whitten 1987; van Noordwijk 1985). All these hypotheses predict a close relationship between swelling size and ovulation. The hypothesis that has received most support to date, however, is the graded-signal hypothesis (Nunn 1999), which states that swellings function as a probabilistic signal of ovulation, indicating when it is most likely but not its exact timing. In particular, it predicts that ovulation will occur during peak swelling, although with some variation. This hypothesis has received support from most studies examining the relationship between ovulation and swelling size, even from those species in which these two variables are less closely timed (bonobos: Reichert *et al.* 2002; chimpanzees: Deschner *et al.* 2003, 2004; long-tailed macaques: Engelhardt *et al.* 2005; Barbary macaques: Brauch *et al.* 2007; Möhle *et al.* 2005). Our results, however, do not show conclusive support for the graded-signal hypothesis. While we show that in Sanje mangabeys there is a large inter- and intraindividual variation in the duration of maximum tumescence—which can vary between 1 and 12 days—and that ovulation can happen any time during that period, we found no cases in which ovulation occurred outside of maximum tumescence (albeit we could not rule out this possibility). Furthermore, the conspicuous nature of the shiny phase, as well as the frequent overlap between its onset and ovulation, suggest that in Sanje mangabey swellings not only are less probabilistic signals of ovulation than in other species, but that in fact they are a relatively accurate indicator of fertility, signaling when ovulation is most likely to occur. As such, we would expect male Sanje mangabeys to use the presence of the shiny phase to reliably assess when females are most fertile.

Given that we used each event as the unit of analysis, most data were not independent, as at least some females would contribute more than once to each variable. When we reran the analyses using an average for each female, most of the results remained unchanged, although in a few instances significant differences became nonsignificant, e.g., cycle length between nonconceptive cycles of adult females vs. adolescents, and vice versa, e.g., maximum tumescence duration between cycles of maximum and high probability of conception combined vs. adolescents. As results were consistent for the majority of cases, however, we are confident that, within the existing limitations of our restricted dataset, using each event and not each female as the unit of analysis did not compromise the findings of this study.

A pattern that clearly stood out in our results was the much longer duration of the shiny phase in adolescents. In other primate species, adolescent females show irregular menstrual cycles (Hartman 1931), as well as exaggerated signals of fertility, such as unusually large sexual swellings that may function as a stimulus for adult males to mate with adolescents (Anderson and Bielert 1994). In our study, in addition to the longer shiny phases, the cycles of adolescent females were also significantly longer compared to cycles of adult females. These cycles were likely anovulatory, as a recent study found adolescent Sanje mangabeys to cycle for >16 mo without conceiving, while parous adult females with a surviving infant typically conceived within five cycles (Fernández *et al.* 2014). This pattern of irregular, infertile cycles and exaggerated displays of fertility matches what has been found in other primates (reviewed in Anderson and Bielert 1994).

As mentioned earlier, the methods we used in this study did not allow for the detection of small-scale changes over the course of one cycle in the tumescence of the sexual swelling, as has been found in some species (Brauch *et al.* 2007; Deschner *et al.* 2004; Higham *et al.* 2008b). These studies used photographic techniques to measure absolute sexual swelling size, which correlated with fE, and continued increasing even within the maximum tumescence period. These methods also detected an increase in swelling size as a female underwent consecutive cycles (Emery and Whitten 2003; Deschner *et al.* 2004; Higham *et al.* 2008b; Huchard *et al.* 2009), with the conceptive cycle displaying the largest sexual swelling (Alberts *et al.* 2006; Daspre *et al.* 2009; Fitzpatrick *et al.* 2014; Gesquiere *et al.* 2007; Higham *et al.* 2012). Moreover, Douglas *et al.* (2016) recently demonstrated that studies using a categorical scale to measure swelling size, like the present study, tended to overestimate the length of the period of maximum tumescence, further masking any relationship that may exist between ovulation and swelling size. Given that in our study we used a categorical scale, we cannot rule out the possibility that swelling size and ovulation are more closely linked than what our results indicated, and that male Sanje mangabeys have an additional morphological cue, other than maximum tumescence and the shiny phase, to distinguish female reproductive state and to pinpoint the time of ovulation. If this were the case, we would expect male Sanje mangabeys to guard females during ovulation, regardless of whether it occurs outside the shiny phase or outside of maximum tumescence.

In conclusion, this study adds to the body of evidence demonstrating that primate sexual swellings can convey information about female fertility and the probability that a cycle would be conceptive. In particular, the onset of the shiny phase typically marked the timing of ovulation. In addition, swellings developed during gestation were less likely to display the shiny phase. Given the conspicuous nature of the shiny phase it seems that in Sanje mangabeys the information conveyed by sexual swellings is more reliable than in many other cercopithecine primates, where swellings tend to indicate the probability of ovulation rather than the exact timing. Further research is needed to better understand what factors are driving the observed interspecific variation in the reliability of sexual swellings as signals of female fertility, as well as the consequences of this variation in males’ and females’ reproductive strategies.

## Electronic supplementary material

ESM 1 (DOCX 907 kb)

Photographs of the sexual skin of female Sanje mangabeys throughout the menstrual cycle (Fig. S1) and information on cycle abnormalities and their presumed correlates (Table SI) are provided in the Electronic Supplementary Material.
