# Supplementary material for: Exaggerated Sexual Swellings and the Probability of Conception in Wild Sanje Mangabeys (Cercocebus sanjei)
Source: Int J Primatol. 2017 Apr 25;38(3):513–32. doi: 10.1007/s10764-017-9961-1 (PMC5487806; doi:10.1007/s10764-017-9961-1)
Supplement: Supplementary file 1 — (DOCX 907 kb) [file 10764_2017_9961_MOESM1_ESM.docx]

**Electronic Supplementary Material**

**Exaggerated Sexual Swellings and the Probability of Conception in Wild Sanje Mangabeys (*Cercocebus sanjei*)**

**David Fernández · Diane Doran-Sheehy · Carola Borries · Carolyn L. Ehardt**

**Fig. S1** Sexual skin of female Sanje mangabeys **(a)** at score 0 or flat, **(b)** score 5 or maximum tumescence, and **(c)** score 6 or the beginning of detumescence. **(d)** Female displaying a sexual swelling in maximum tumescence.

**(a)** **(b)**


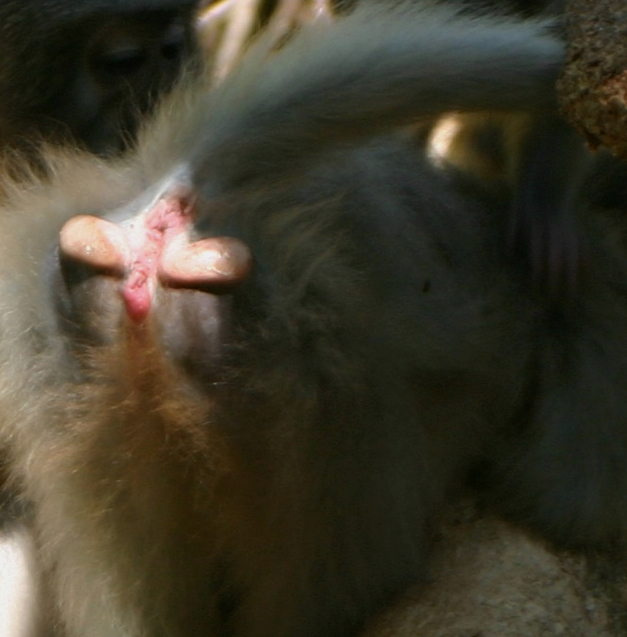

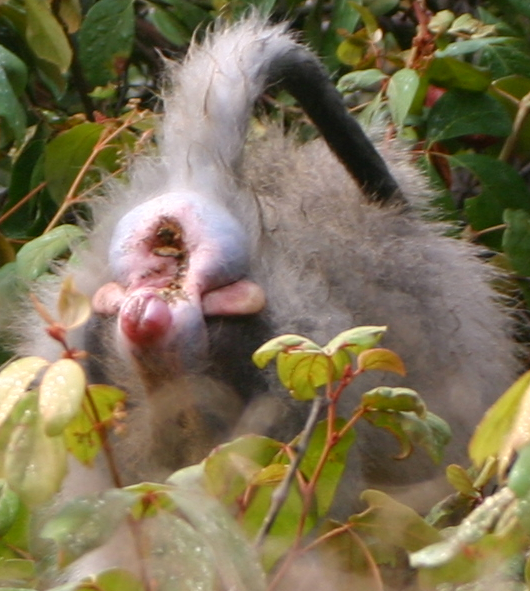


**(c)** **(d)**


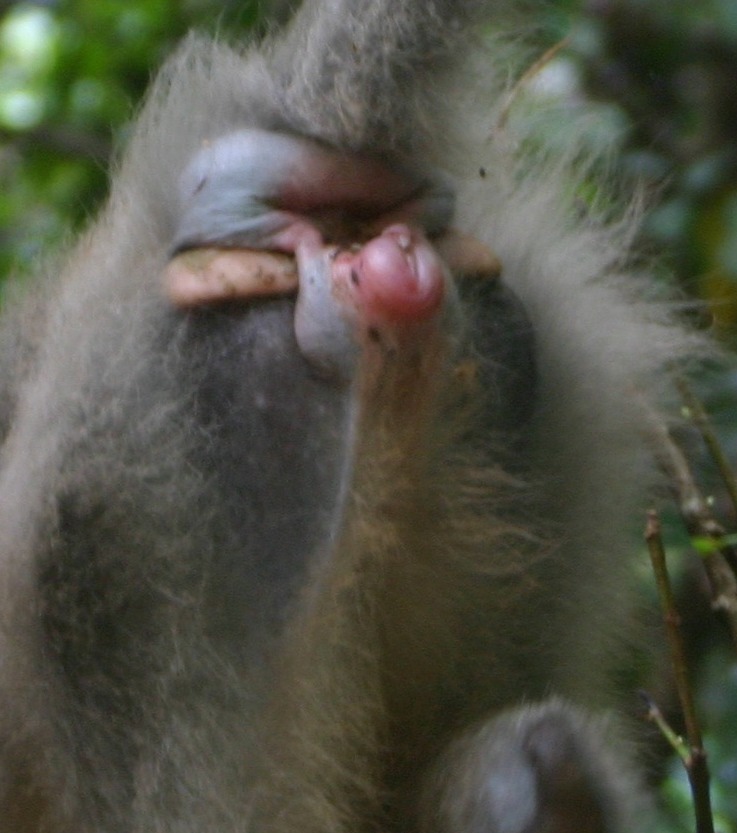

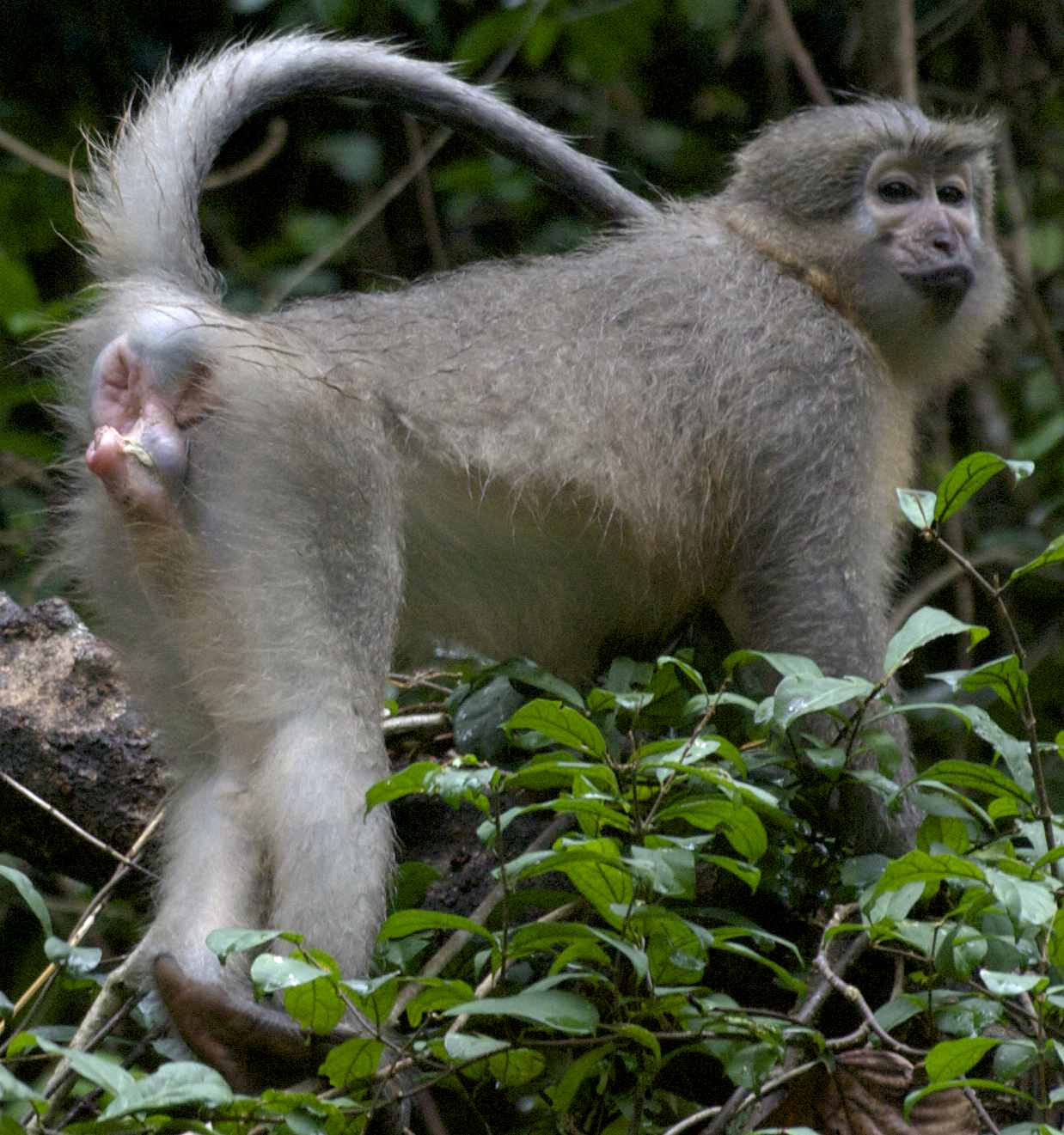


**Table SI** Cycle anomalies observed and their presumed correlates for female Sanje mangabeys

| Cycle anomaly | Female ID | Duration (days) | Presumed correlate |
| --- | --- | --- | --- |
| Long inflation | mdo | 93 | Unknown |
| Long inflation | kum | 60 | Unknown |
| Does not reach MAX | mzu | n/a | First cycle after surviving infant |
| Does not reach MAX | yey | n/a | First cycle after death of 41-day old infant |
| Does not reach MAX | uvi | n/a | Second cycle after surviving infant |
| Long tumescence after MAX | bad | >22 | Returning to group after an absence of 31 days |
| No shiny phase | uvi | n/a | First cycle after surviving infant |
| No shiny phase | yey | n/a | First cycle after death of the infant |

None of these cycles were used in the calculations of the characteristics of the sexual swellings.
